# Diversity and bioactivities of fungal endophytes from *Distylium chinense*, a rare waterlogging tolerant plant endemic to the Three Gorges Reservoir

**DOI:** 10.1186/s12866-019-1634-0

**Published:** 2019-12-10

**Authors:** Xiaoxiang Duan, Fangfang Xu, Dan Qin, Tiancong Gao, Weiyun Shen, Shihao Zuo, Baohong Yu, Jieru Xu, Yajun Peng, Jinyan Dong

**Affiliations:** 1 Chongqing Key Laboratory of Plant Resource Conservation and Germplasm Innovation, School of Life Sciences, Southwest University, Chongqing, 400715 People’s Republic of China; 2 Laboratory Animal Research Institute of Chongqing Academy of Chinese Materia Medica, Chongqing, 400065 People’s Republic of China; 3 First Affiliated Hospital, Huzhou Teachers College, The First People’s Hospital of Huzhou, 158 Guangchanghou Road, Huzhou, 313000 People’s Republic of China

**Keywords:** *Distylium chinense*, Bioactivity, Endophytic fungi, Identification, Metabolites

## Abstract

**Background:**

The present study involves diversity and biological activities of the endophytic fungal community from Distylium chinense, a rare waterlogging tolerant plant endemic to the Three Gorges Reservoir. This study has been conducted hypothesizing that the microbial communities in the TGR area would contribute to the host plant tolerating a range of abiotic stress such as summer flooding, infertility, drought, salinity and soil erosion etc., and they may produce new metabolites, which may possess plentiful bioactive property, especially antioxidant activity. Therefore in the current study, the antioxidant, antimicrobial and anticancer activities of 154 endophytes recovered from D. chinense have been investigated. Furthermore, the active metabolites of the most broad-spectrum bioactive strain have also been studied.

**Results:**

A total of 154 fungal endophytes were isolated from roots and stems. They were categorized into 30 morphotypes based on cultural characteristics and were affiliated with 27 different taxa. Among these, the most abundant fungal orders included Diaporthales (34.4%) and Botryosphaeriales (30.5%), which were predominantly represented by the species *Phomopsis* sp. (24.7%) and *Neofusicoccum parvum* (23.4%)*.* Fermentation extracts were evaluated, screening for antioxidant, antimicrobial and anticancer activities. Among the 154 isolates tested, 99 (64.3%) displayed significant antioxidant activity, 153 (99.4%) exhibited inclusive antimicrobial activity against at least one tested microorganism and 27 (17.5%) showed exclusive anticancer activity against one or more cancer cell lines. Specifically, the crude extract of *Irpex lacteus* DR10–1 exhibited note-worthy bioactivities. Further chemical investigation on DR10–1 strain resulted in the isolation and identification of two known bioactive metabolites, indole-3-carboxylic acid (**1**) and indole-3-carboxaldehyde (**2**), indicating their potential roles in plant growth promotion and human medicinal value.

**Conclusion:**

These results indicated that diverse endophytic fungal population inhabits D. chinense. One of the fungal isolate DR10–1 (*Irpex lacteus*) exhibited significant antioxidant, antimicrobial and anticancer potential. Further, its active secondary metabolites **1** and **2** also showed antioxidant, antimicrobial and anticancer potential.

## Background

Endophytic fungi in plants are microorganisms that parasitize symbiotically in the internal tissues during the whole or part of their life cycles of the hosts without causing apparent pathogenic symptoms [1], but may turn pathogenic during host senescence [2]. Accumulated evidence has confirmed that plant endophytes from special or extreme environment has many effects on host ecological adaptability [3*–*5]. It is well known that the concurrence of endophytes may accelerate plant growth and increase the survival rate of biotic or abiotic stresses, such as plant diseases, pests, drought, salinity and extreme temperatures [6*–*9]. Specifically, some endophytes are beneficial to plants by producing special substances, such as secondary metabolites, which can prevent the host from being attacked successfully by fungi and pests [10]. So far, endophytes, especially those under complex and extreme conditions, have been shown to produce a variety of metabolites with complex structures, such as alkaloids, terpenoids, polyketides, lipids, glycosides, isoprenoids, and hybrids of those metabolites, etc. [11*–*13]. More interestingly, these metabolites also showed a variety of interesting bioactivities including antifungal [14], antibacterial [15], anticancer [16], anti-HIV [17], antioxidants [18], etc. Due to these, endophytes from an untapped diverse habitat are a significant source of novel and natural drugs [19].

After Three Gorges Dam is constructed, the Three Gorges Reservoir (TGR) forms a new vast hydro-fluctuation belt with an elevation of 145 m in summer to 175 m in winter, a length of more than 2000 km and an area of 300 km^2^ [20, 21], which has provided unique ecological habitats for those diverse species in the TGR area [22]. Many field surveys have shown that most of the pre-dam riparian vegetation is gradually dying out due to the inability to adapt to the reversal of submergence time, the prolongation of flood duration and the new hydrological fluctuation zone (up to 30m in elevation) [23]. Generally, plants use limited oxygen and light under flood conditions, resulting in production of excessive reactive oxygen species (ROS) [24], which were the key factors that hindered the growth and development of submerged plants [25, 26]. They are forced to undergo the oxidative pathway [27], and usually develop an antioxidant defense system consisting of some antioxidant enzymes and specific metabolites to convert these excessive ROS into harmless products in order to protect themselves [28, 29].

As symbionts, endophytic fungi can produce antioxidants, block the chain reaction of ROS to help host plants respond to various biotic and abiotic stresses [9, 30]. Some studies have also showed that endophytes can increase the survival rate of host plants during flooding stress by producing antioxidants independently [31, 32]. Severe oxidative damage of free radicals has been confirmed to be associated with various diseases, including cancer, inflammation, aging and neurodegenerative diseases [33]. It has been advised that antioxidants should be warranted in the enhancement of human health [34, 35]. Currently, the demand for natural antioxidants from endophytic fungi has been increasing along with the finding that natural antioxidants have fewer side effects on human health than artificially synthesized substances [36, 37]*.* Additionally, the search for safer and novel drugs based on the natural product from endophytes is of utmost importance because of the increasing incidence of cancer and the recently emerged, rapid evolution of superbugs due to antibiotic resistance [38, 39].

**Fig. 1:**
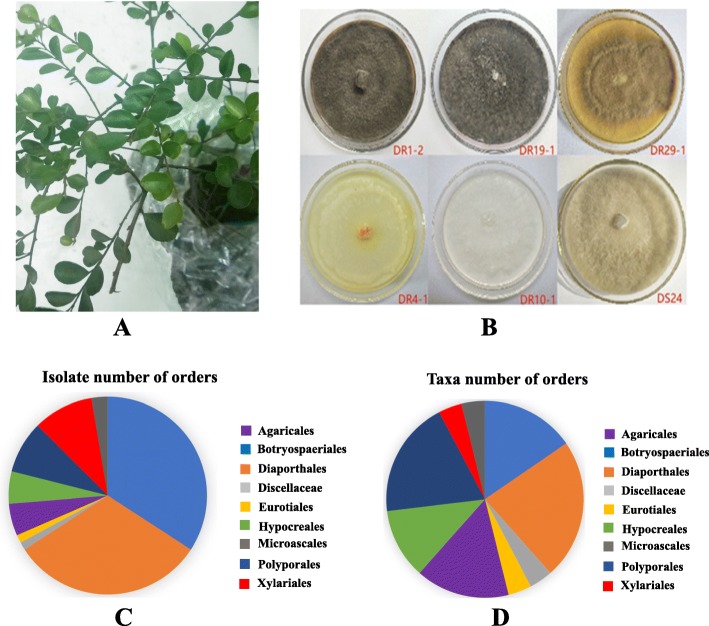
*D. chinense* plant and taxonomic distribution of endophytic fungi. (**a**) *D. chinense* plant. (**b**) Representative fungal morphotypes isolated from *D. chinense* growing on potato dextrose agar (PDA) for one week at 26 °C. (**c**) Distribution of fungal isolates (*n* = 154) belonging to each order (*n* = 9). (**d**) Distribution of fungal taxa (*n* = 27) belonging to each order (*n* = 9)

After Three Gorges Dam is constructed, many abiotic stresses in the natural habitat strongly influence plant growth and development, such as summer flooding, infertility, drought, salinity and soil erosion etc. So far, only a few highly tolerant plants have been reported to survive, which include *Salix variegate*, *Morus alba L.*, *Myricaria laxiflora* [22, 40]. Among them, *Distylium chinense* (Fr.) Diels, a rare evergreen ornamental shrub of Hamamelidaceae family known for the beautiful flowers (Fig. 1a), is a native species to the riparian wetland in the TGR area of the Yangtze River and its tributaries [20, 41, 42]. Since 2005, *D. chinense* was considered as an ideal choice for solid embankment after the construction of the Three Gorges Dam owing to its strong root system, erosion tolerance, strong flooding tolerance and resistance to sand burial soaks [43]. Several biological studies have been made for *D. chinense* such as morphological characteristics, natural habitat, genetic diversity, community structure, ecological adaptability, reproductive allocation and propagation methods [42, 44, 45]. It should be noted that the roots of *D. chinense* has been used in traditional Chinese medicine and folk medicine as an analgesic, antirheumatic and diuretic [46]. However, there is no information on the diversity and bioactive potential of endophytes community from *D. chinense*. Thus, the aim of this study was to provide the first evidence of endophytic fungi diversity within the *D. chinense*, provide a working collection of endophytes and investigate endophytes with antioxidant, antimicrobial and anticancer activities in order to explore the potential sources of novel drugs.

## Methods

### Plant material

Three healthy and asymptomatic *D. chinense* plants were randomly collected from different locations on an island in the Banan district (N29º42'45.63", E106º60'69.43") of Chongqing of China in the Three Gorges Reservoir area in October 2014. All plant materials were immediately sent to the laboratory and stored in a refrigerator at 4°C. Each sample tissues were used within 24 h after collection. The plant samples were identified as *D. chinense* by Prof. Hongping Deng and were preserved in Chongqing Key Laboratory of Plant Resource Conservation and Germplasm Innovation, School of Life Science, Southwest University, Chongqing 400715, China.

### Isolation and cultivation of endophytic fungi

The surface sterilization and isolation of fungal endophytes were carried out, and some improvements were made [47]. In the first instance, all stems and roots of plant materials were thoroughly washed in running tap water to remove debris and then air-dried naturally in the clean bench. Clean tissue pieces were disinfected in series of solutions: 75% ethanol; sterile distilled water; 0.1% mercuric chloride (HgCl) (v/v). Finally, they were again rinsed with sterile distilled water three times. After surface sterilization, the tissues were dried on blotting sheets, cut into 0.5 cm lengths and transferred to potato dextrose agar (PDA) medium supplemented with 60 mg/mL of streptomycin and 100 mg/mL of ampicillin using an aseptic technique to inhibit the bacterial growth. At the same time, the final sterile water used for washing the tissues (100 *μ*L) was also plated on the PDA to confirm the sterilization effect of the surface. The inoculated plates were incubated at 28°C in darkness for 2-15 days to allow the growth of endophytic fungal hyphae and checked regularly. Pure isolates were checked for purity and transferred to another PDA plate by the hyphal tip method [48]. The obtained endophytic fungal isolates were coded according to their source tissues (DR1-1, DR1-2, DR2-1, etc. from roots and DS2-1, DS3-1, DS1-2, etc. from the stems). These endophytes were classified according to colony color, form, elevation and margin characteristics on PDA. Based on the groupings, strains with different morphology were screened for molecular identification.

### Molecular identification and phylogenetic evaluation of endophytic fungi

According to the above simple classification, each type of fungi was chosen as the representative for molecular biological identification using the fungal genomic deoxyribonucleic acid (DNA) extraction. Fungal genomic DNA extraction was previously described by Landum et al. according to the manufacturer’s instructions using the DNeasy Plant Minikit (Qiagen, Germany) [49]. The nuclear ribosomal DNA internal transcribed spacer (ITS) of the fungal isolates were amplified by forward primer, ITS1-F (5′-TCCGTAGGTGAACCTGCGG-3′) and reverse primer, ITS4 (5′-TCCTCCGCTTATTGATATGC-3′) [50]. The final reaction volume was 25 *μ*L, containing 12.5 *μ*L of 2X PCRBIO Taq Mix Red (PCR Biosystems, UK), 0.4 *μ*M of forward and reverse primers and 10 ng of genomic DNA template. For negative control, the DNA was replaced with distilled water to verify absence of contamination. PCR was carried out using MyCycler ^TM^ (Bio-Rad, USA), programmed for 5 min 94°C; 30 cycles for 30s at 94°C, 60s at 55°C, and 1min at 72°C; and a final 10 min extension at 72 °C. The PCR products were separated using 1% agarose gel in 1X TAE buffer (90mM Tris-acetate and 2 nM EDTA, pH 8.0), with ethidium bromide (0.5*μ*g/mL) staining and recorded with FluorChemTM (Alpha Innotech, USA). The PCR products were sequenced by Invitrogen Co. Shanghai.

In phylogenetic evaluation, the ITS DNA sequences and downloaded sequences of their nearest neighbors were aligned in Alignment Explorer of MEGA 4 software using ClustalW option [51, 52]. MUSCLE (UPGMA) algorithm was used to prune and verify the sequence. The evolutionary distances and history were calculated by using the neighbor-Joining methods [53]. The robustness of the trees were assessed by bootstrap analysis with 1000 replication [54].

### Bioactivity evaluation

#### Fermentation and preparation of fungal extract

Fermentation and preparation of the fungi were determined according to the scheme proposed by Ya-Li et al. with some modifications [55]. Briefly, all isolates were cultured in potato dextrose broth (PDB, the medium contained potato 200 g and glucose 20 g in 1 L of purified water) for 14 d at 28 °C on a shaker at 180 r/min. Crude fermentation broth was filtered with eight layers of gauze. Filtered liquid was extracted three times with the same amount of ethyl acetate. The organic solvent extract was then evaporated under reduced pressure to yield an ethyl acetate extract. The ethyl acetate extracts were dissolved in methanol and the final concentration was 10 mg/mL for bioactivity screening.

#### Antioxidant activity

The radical scavenging ability was evaluated by using adapted 2,2’-diphenyl-b-picrylhydrazyl (DPPH) method described previously with some modification [56]. Thus, an aliquot of extract (50 *μ*L) was added to 150 *μ*L of methanol DPPH (50 *μ*M). The reaction mixture was transferred to a 96-well microtitre plate and incubated at room temperature for 30 min in the dark and absorbance was measured at 517 nm using a microtiter plate reader (Bio-Rad 680, BIO-RAD, USA). Ascorbic acid (Vc) and methanol were used as positive and negative controls, respectively. Meanwhile, three experimental replicates were taken for the assay.

#### Antimicrobial activity

The determination of antimicrobial activity was based on the disk diffusion method with some modification [57]. Each disc (Oxford cup, 6 mm diameter) contained 200 *μ*g of endophytic fungi extraction (10 mg/mL). The indicator organisms included gram-negative: *Escherichia coli* (ATCC25922, EC), *Pseudomonas aeruginosa* (CMCC(B)10104, PA); gram-positive: *Staphylococcus aureus* (ATCC6538, SA), *Bacillus subtilis* (ATCC6633, BS); three pathogenic fungi *Penicillium* (ATCC9080, P), *Aspergillus niger* (CMCC(F)98003, AN) and *Candida albicans* (CMCC(F)98001, CA). There were purchased from Shanghai Luwei Technology Co., Ltd. Streptomycin and amphotericin B were used as positive controls and methanol as negative control. The antimicrobial activities were determined according to diameters of inhibitory zones (ZI) and experiments were repeated three times.

#### Anticancer activity

Human papillary thyroid carcinoma cell line IHH4 and human pancreatic adenocarcinoma cell line CFPAC-1 were obtained from the Cell Line Bank of the Chinese Academy of Science. The anticancer activity was determined according to CCK-8 assay [58]. Cisplatin was used as the positive control and repeated for three times.

#### Isolation of bioactive metabolites

Based on the results of the above antioxidant, antimicrobial and anticancer activities, the strain *Irpex lacteus* DR10-1 was selected for the chemical analysis because it exhibited widest broad-spectrum bioactivities. *Irpex lacteus* DR10-1 culture filtrate 14L was fermented by the same method as above mentioned. Crude ethyl acetate (EtOAc) extracts from *Irpex lacteus* DR10-1 (6.7g) was obtained and further purified by a silica gel column (200-300 mesh, 4.0 × 70 cm, with 70 g of silica gel), and eluted with gradient mixtures of petroleum ether (60-90 °C) and EtOAc to yield 5 fractions (A1-A5). Fraction A2 (156 mg) was further purified by a silica gel column chromatography (300-400 mesh, 2.0 × 25 cm, with 15 g of silica gel) and eluted with gradient mixtures of chloroform (CHCl_3_) and EtOAc to yield compound **1** (30mg). Fraction A4 (98 mg) was further purified by a silica gel column chromatography (300-400 mesh, 1.0 × 25 cm, with 35 g of silica gel), and eluted with gradient mixtures of CHCl_3_ and methanol (MeOH) to obtain compound **2** (25mg).

Nuclear magnetic resonance (NMR) spectra were recorded by Bruker Ascend 500 spectrometer. The spectrometer operated at 500 MHz for ^1^H nuclei and 125 MHz for ^13^C nuclei. Chemical shift was quoted in parts per million (ppm), referring to the appropriate residual solvent peak.

#### Statistical Analysis

Using species as the statistical unit, the number of isolates (*N*) and the isolation frequency (*IF*) for each endophytic fungal species in different tissues or the total plant (Additional file 1 Table S1) were calculated. Species richness index (*S*) and Margalef index (*D´*) were used to evaluate species richness, which were two important parameters for alpha diversity analysis [59]. Shannon-Wiener index (*H´*) and Simpson’s diversity index (*D*s) were used to the species diversity, respectively [60, 61]. Additionally, the Jaccard Similarity Index (*JC*) was used to compare the species composition of the stem and root tissues [62]. Results were expressed as mean ± standard deviation (SD) of triplicate of measurements for the DPPH and CCK-8 assays. Data were conducted with SPSS 18.0 for Windows (SPSS Inc., Chicago, USA).

## Results

### Community composition and abundance

**Fig. 2:**
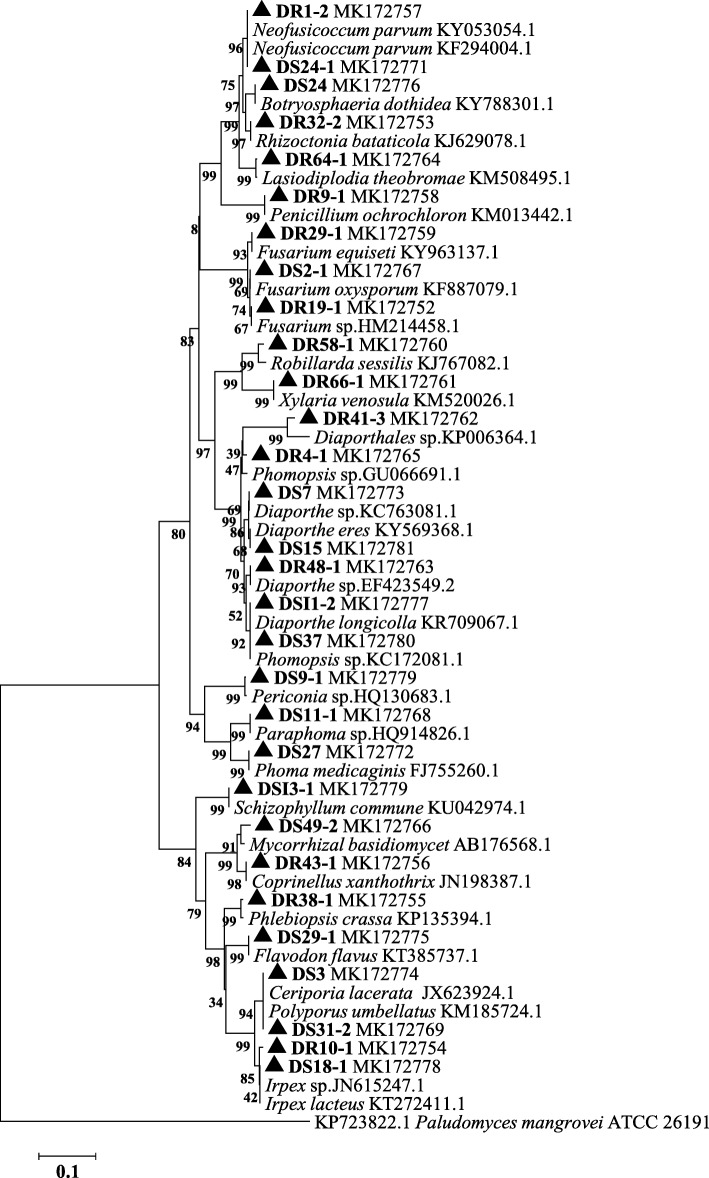
Phylogeny analyses of endophytic fungi from *D. chinense*. The tree was derived by neighbor-joining methods analysis of ITS1–5.8S-ITS4 sequences [53] and 30 sequences retrieved from Gen Bank. The percentage of replicate trees in which associated taxa were clustered together in the bootstrap test (1000 replicates, values below 50% are not shown) are shown next to the branches. Phylogeny analyses were conducted in MEGA 4 software [51, 52]

A total of 154 fungal endophytes were isolated from *D. chinense* plants collected from the TGR area. Among them, 30 different representative morphospecies were determined according to cultural characteristics (Fig. 1b). Of these detected, 30 isolates were categorized into 27 different taxa (Ascomycota, 19; Basidiomycota, 8), and further into nine distinct orders (Fig. 1c). The Fig. 2 showed the phylogenetic tree of 30 fungal strains isolated from the NCBI database and the accession numbers of the matched rDNA-ITS sequences. The supplementary table data (Additional file 1: Table S1) provided detailed information on 30 representative strains, including their sources and isolation frequencies.

At the order level, the Diaporthales possessed the most taxa, six taxa, accounting for 22.2% of the total fungal taxa and they had 48 isolates, around 31.2% of the total fungal isolates (Fig. 1d). Conversely, the Botryospaeriales had the most isolates, 52 isolates, accounting for 33.8% of the total fungal isolates and they possessed four species, around 14.8% of the total fungal species. The Polyporales and Agaricales were the second and third most abundant orders with high species, and together constituted approximately 33.3% of all the species. Analogously, the Xylariales and Polyporales were the second and third most abundant isolates, and together constituted approximately 18.1% of all the isolates. The other identified orders were the Hypocreales, Microascales, Eurotiales and Discellaceae, which together constituted approximately 22.2% and 10.4% of all species and isolates, respectively (Fig. 1d). Interestingly, the most common fungal species between roots and stems were *Phomopsis* sp. (24.7%), followed by *Neofusicoccum parvum* (23.4%). However, *Phomopsis* were not from order with highest isolate rates.

### Species diversity and richness abundance of fungi

**Fig. 3:**
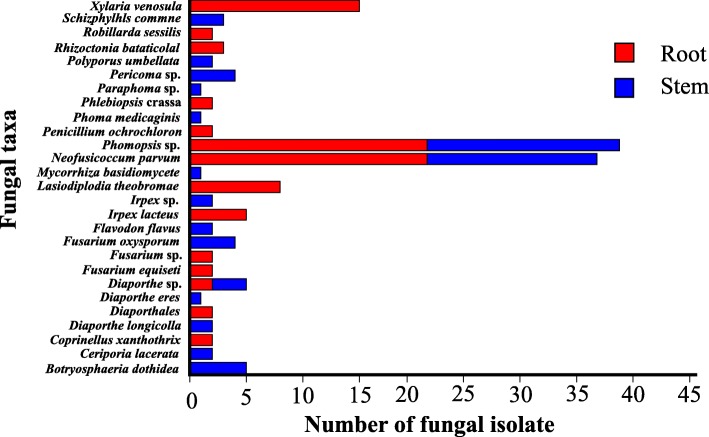
Distribution of the fungal isolates (*n* = 154) across different plant tissues

**Fig. 4:**
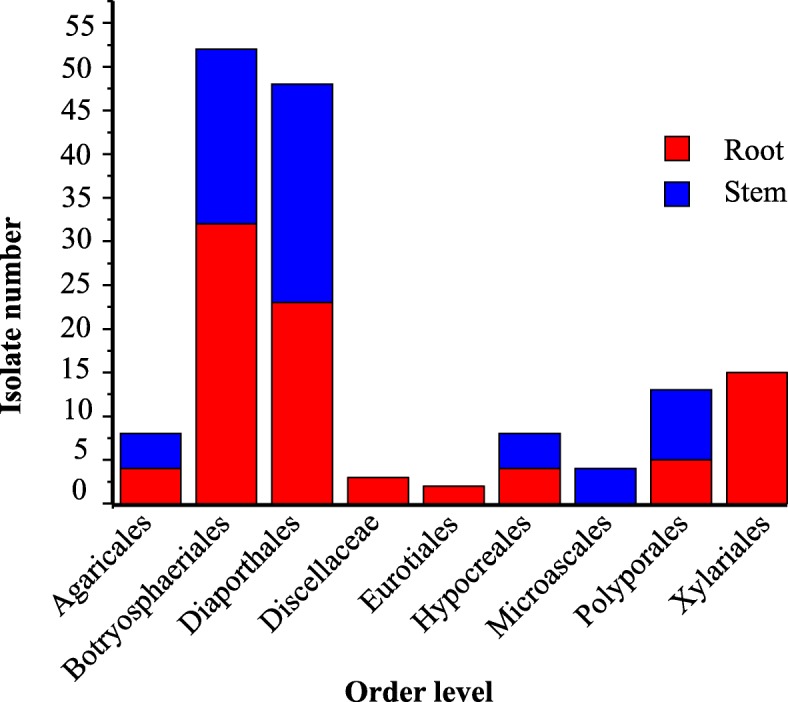
Distribution of the orders of the fungal isolates (*n* = 154) from different tissues

The richness and species diversity of culturable endophytic fungi were significantly higher in stems than in roots (Table 1). Among the 27 total taxa, 16 (59.3% of total) were obtained from the stems. A total of 3 fungal taxa- *Neofusicoccum parvum*, *Phomopsis* sp. and *Diaporthe* sp. were distributed in both plant tissues, but ten taxa-*Fusarium* sp., *Fusarium equiseti*, *Xylaria venosula*, *Lasiodiplodia theobromae*, *Penicillium ochrochloron*, *Rhizoctonnia bataticola*, *Robillarda sessilis*, *Coprinellus xanthothrix*, *Polyporus crassa* and *Irpex lacteus* were only found in the roots (Fig. 3). Similarly, of the nine orders, two were found in both stems and roots, but the Hypocreales, Xylariales, Eurotiales and Discellaceae were unique to the roots (Fig. 4). Additionally, Shannon-Wiener index (*H'*) and Simpson diversity index (*Ds*) could be used to analyze species diversity. Generally, the higher the Shannon’s diversity index (usually between 1.5 and 4.5), the closer the Simpson’s diversity index is to 1, the stronger the adaptability of the community to the change of micro environment is, and the community presents the trend of expanding the distribution range and entering the new environment [63]. On the other hand, the species richness (*S*) and Margalef index (*D'*) can reflect the richness of endophytic fungi species. The larger the values of *S* and *D'* were, the richer the species of endophytic fungi were [64]. As shown in Table 1, the species richness and diversity of endophytic fungi in stems were higher than those in roots, and the values of *S* (16), *D'* (3.5802), *H'* (2.5323) and *D*s (0.8659) were higher. In addition, the similarity index (Jaccard's index) was used to estimate the similarity between stem and root. Although stem and root samples collected in TRG field were adjacent to each other and lived in the same place, the Jaccard's index only showed 0.11 between stems and roots, showing low similarity. These indices showed that endophytic fungi in different tissues had significant diversity.

**Table 1:** Diversity analyses of endophytic fungi from *D. chinense*

Diversity Index	Different Tissues		Total
	Root	Stem	
Species richness (*S*)	14	16	27
Margalef index (*D´*)	2.9109	3.5802	5.1619
Shannon-Wiener index (*H´*)	2.1828	2.5323	2.4824
Simpson diversity index (*Ds*)	0.8366	0.8659	0.8646
Jaccard’s indice (*JC*)		0.11

### Bioactivity evaluation of fungal endophytes

**Fig. 5:**
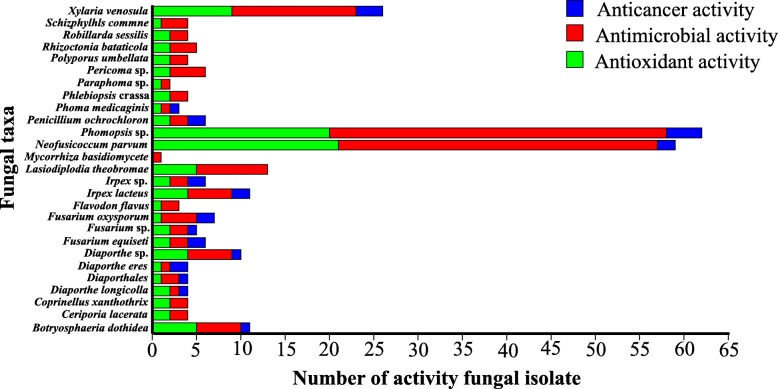
Distribution of the activity fungal isolates

As mentioned above, one of the main purposes of this study was to identify endophytic fungi that could be cultured and applied to develop their potentially beneficial properties for plants and humans. All 154 fungal endophytes isolated from *D. chinense* at TGR were evaluated for their antioxidant, antimicrobial and anticancer activities (Additional file 1: Table S2-S4). Among the 154 isolates, 99 (64.3%), 153 (99.4%) and 27 (17.5%) fungal extracts showed antioxidant activity, antimicrobial activity against at least one indicator organisms and anticancer activity against one or two human cancer cell lines, respectively. Among the isolates that displayed the individual activities, *Phomopsis* sp. accounted for 20, 38 and 4 of isolates possessing antioxidant, antimicrobial and anticancer activities, respectively (Fig. 5). *Neofusicoccum parvum* and *Xylaria venosula* were also enriched in isolates showing bioactivities. However, in our assay, the isolates belonging to *Mycorrhiza basidiomycete*, did not display antioxidant and anticancer activities (Fig. 5). Thus, the distribution of active strains showed obvious taxonomic specificity. Interestingly, the fungal extracts of DS16-1 (*Phomopsis* sp.), DR10-1 (*Irpex lacteus*), DS9-1 (*Periconia* sp.) and DS6 (*Phomopsis* sp.) showed higher antioxidant activity than that of ascorbic acid, acted as a scavenger of DPPH radical with IC_50_ values of 2.59 ± 0.03, 2.79 ± 0.04, 2.95 ± 0.03 and 2.97 ± 0.01 *μ*g/mL, respectively. For the antimicrobial activity, fungal extract of DR28-1 (*Phomopsis* sp.) displayed the highest antimicrobial activity against *Pseudomonas aeruginosa* with a zone of inhibition (ZI) value of 40 mm, fungal extract of DS35-1 (*Ceriporia lacerta*) showed the highest antimicrobial activity against *Staphylococcus aureus*, with a ZI value of 40 mm, the fungal extracts of DR41-2 (*Ceriporia lacerta*) had the highest activity against *Aspergillus niger*, and its ZI value was 30 mm. Particularly, the extract DR10-1 (*Irpex lacteus*) was the only strain that exhibited broad antimicrobial capability because it inhibited the growth of all tested pathogens. As for anticancer activity, fungal extract of DR46-1 (*Phomopsis* sp.) showed the highest anticancer activity against IHH4 cell line with IC_50_ values of 9.20 ± 0.02 *μ*g/mL.

### Characterization of metabolites of strain DR10-1

**Fig. 6:**
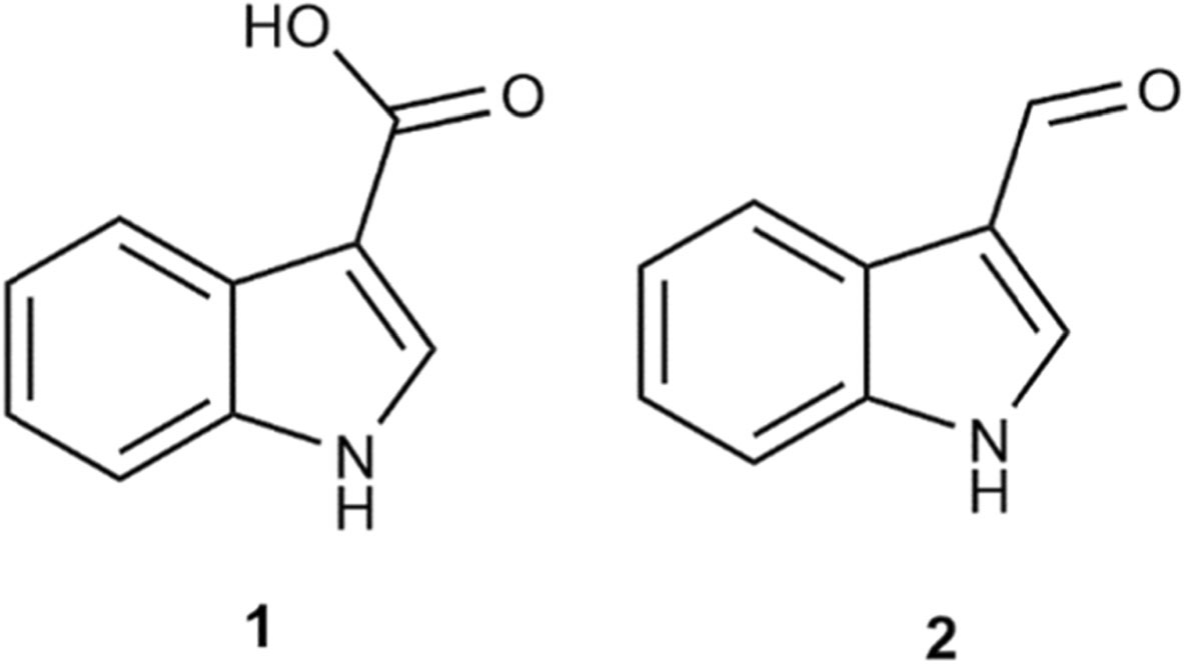
The chemical structure of compounds **1–2**

Among 154 strains recovered from *D. chinense*, the EtOAc extract of the culture broth of *Irpex lacteus* DR10-1 (Additional file 2: Figure S1-S2) exhibited higher antioxidant activity than that of ascorbic acid, antimicrobial capability by inhibiting the growth of seven tested pathogens and showed anticancer activity against both tested cancer cell lines, and was subjected to column chromatography over silica gel, Seqhadex LH-20 to afford two known compounds. The structures of the two known compounds were established as indole-3-carboxylic acid (compound **1**) [65] (Additional file 2: Figure S3-S4) and indole-3-carboxaldehyde (compound **2**) [66] (Additional file 2: Figure S5-S6) by comparing their spectroscopic data with those in the literature (Fig. 6)

## Discussion

Considering the roles of endophytic fungi in plant development, growth, adaptability and diversity, we needed to fill this gap in order to exploit of endophytes for a better understanding of *D. chinense* plant and their important metabolites found in the TGR. Therefore, one of the purposes of this study was to examine the community composition of fungal endophytes from TGR. Here, we took a culture-dependent approach, since our final goal was to build a working collection of fungal endophytes that could be explored for their potentially beneficial properties in *D. chinense* plant. In this work, a total of 154 endophytic fungi were isolated from D. chinense in the TGR and classified into 27 different taxa according to their morphological characteristics and unique phenotypic characters. The identified fungi were mainly composed of Phomopsis, N. parvum, Diaporthe, Fusarium and Irpex with relative frequencies 24.7%, 23.4%, 3.2%, 5.2% and 4.5%, respectively. Among them, fungi that belong to Phomopsis, Diaporthe, Fusarium and Irpex have been reported as the main endophytes of wetland shrub Myricaria laxiflora in the TGR [67] and riparian plant species [68]. Additionally, Fusarium, Phomopsis and Irpex has also been reported to be not sensitive to flooding stress [67]. By contrast, other several genera, including Penicillium ochrochloron, Mycorrhizal basidiomycete, Ceriporia lacerta, Diaporthe longicolla, Diaporthe eres, Flavodon flavus, Irpex sp., Parphoma sp. and Phoma medicaginis, were only isolated from D. chienense with low relative abundance. Even so, the existence of these minor genera has been demonstrated to play an important ecological role in their host plants as reported [69]*.*

According to the literatures, fungal endophytic community of land plant mainly belonged to Sordariomycetes, Dothideomycetes and Pezizomycetes fungi while plants from water or moist environments were more often parasitized by Eurotiomycetes [70–72]. In the current study, the most prevalent class was Sordariomycetes with relative frequency of 50%, followed by Dothideomycetes and Eurotiomycetes at 33.8% and 1.3%, respectively. Obviously, both terrestrial and aquatic fungi are present in the *D. chinense* plant. This was in accordance with the report by Kandalepas et al., who discovered high numbers of Sordariomycetes and low numbers of Dothideomycetes and Eurotiomycetes in wetland plants from Louisiana [71].

It has been reported that dark septate endophytes (DSE) could be important as (latent) saprobes, playing a role in host nutrition through complex substrate degradation, and might help to degrade organic matter in nutrient-poor soils in a similar way as ericoid mycorrhizal fungi-mutualistic symbionts that benefit the host plant by mobilizing complex substrates in nutrient poor environments [73]. Here, 7 taxa out of 27 taxa detected were found to be darkly pigmented with thickly walled septate hyphae that can be classified into dark septate fungi, which include *Diaporthales*, *Phomopsis* sp., *Lasiodiplodia theobromae*, *Neofusicoccum parvum*, *Irpex lacteus*, *Periconia* sp., *Botryosphaeria dothidea* [74]. According to our statistics, 20 (13.0%) from 154 isolates belong to this group. In our previous paper, *Salix variegata*, another waterlogging tolerant plant in the TGR area, has also been found to be colonized by abundant DSEs [75]. This was in accordance with the report on the occurrence of DSEs in wetland plant species [76, 77].

Among these isolates found in *D. chinense*, many from the genera *Phomopsis*, *Fusarium*, *Diaporthe, Neofusicoccum parvum*, *Xylaria venosula*, *Lasiodiplodia theobromae* and *Botryosphaeria dothidea* have been reported as common pathogenic fungi in some wild and cultured plants [75]. For examples, *Diaporthe* and *Phomopsis* complex were the causes of seed decay and cause soybean blight and canker diseases [78]; *Neofusicoccum parvum* was reported as one of the most aggressive causal agents of the trunk disease Botryosphaeria dieback [79]; *Botryosphaeria* and its anamorph complex were particularly important for symptoms such as fruit rot, shoot blight, dieback and canker of numerous woody hosts [80]. Although the symptoms of disease did not appear in *D. chinense* plant collected, as reported, these fungi might switch their lifestyles from a mutualistic to parasitic interaction which depended on genetic factors of both partners [81], imbalance in nutrient exchange [82] and environmental variations [83, 84]. Furthermore, the interaction type between an endophyte and a host plant also could be modulated if the plant was subjected to physiological stress [85]. It has been shown that individual fungal species which could switch lifestyles might represent an evolutionary transition, or simply fungi that had achieved remarkable ecological plasticity, might ensure the optimal growth and reproduction in a variety of hosts, which ultimately would lead to the expansion of their bio-geographic distribution [81]. As a whole, mutualistic interactions between fungal invaders and host plants are deciphered as a balance, which is considered as a combination of environmental and physiological effects that benefit both sides [82]. Fitness benefits conferred by mutualistic fungi contribute to or are responsible for plant adaptation to biotic and abiotic stress [86, 87].

Another objective of this study was to assess the potentially beneficial properties of endophytic fungi to humans. All the endophytes extracts were screened for antioxidant, antimicrobial and anticancer activities and they showed at least one biological activity. Among the screened isolates, 99 (64.3%) isolates exhibited remarkable antioxidant activity, of which 18 (11.7%) had very notable activity with IC_50_ value of ≤ 3 *μg/mL*, suggesting that it may protect D. chinense from oxidative stress in the flooding environment as suggested by Zeng et al [88]. Because of the protective effect of antioxidants, they are essential for plant survival and fitness and presumably selection have leaded to both redundant and highly specific pathways that address ROS production and stress mediation [89]. For example, Mirzahosseini et al. have reported that endophytic fungi can alleviate the oxidative damage produced by ROS accumulation in plant cells such as *F. arundinacea* [90, 91]. Regarding antimicrobial activity, 31.2%, 11.7%, 19.5%, 69.5% and 29.9% extracts of endophytes showed activity against *Penicillium, Candida albicans, Aspergillus niger, Staphylococcus aureus* and *Escherichia coli* respectively, which was comparable and even exceeded some results reported by other authors in similar studies [92, 93]. For example, from the 39 endophytic fungal extracts of *Viguiera arenaria* and *Tithonia Diversifolia* plants, Guimaraes et al. found only 5.1% and 25.6% extracts to be active against *Staphylococcus aureus* and *Escherichia coli* respectively [94]. Unexpectedly, *Pseudomonas aeruginosa* was most sensitive to the fungal extracts among the tested bacterial though it was reported to be drug resistant towards many antibiotics [95]. Usually, the fungal extracts also showed higher activity against the Gram-negative than the Gram-positive ones. This different sensitivity has been suggested to be attributed to the high level of lipopolysaccharides that are contained in the Gram-positive bacteria membrane, which could make the cell wall impermeable to bioactive compounds [96]. As for anticancer activity, 27 out of 154 fungal exacts (17.5%) showed activity against IHH4/CFPAC-1 cell line, in which 11 fungal extracts were active against both tested cell lines. Statistically, 18 out of 27 anticancer isolates were exclusively isolated from the roots, 9 were only recovered from stems. Generally, for the same fungal species e.g. Neofusicoccum parvum, the isolates from roots showed stronger bioactivity compared to those from the stems regardless of antimicrobial, antioxidant or anticancer bioactivities. Such data well supported the traditional practice of native people who often used the extracts from roots to relieve analgesic, antirheumatic and diuretic [43]*.*

Of these isolates screened, a high proportion of bioactivities were mostly detected from the fungal extracts belonging to *Phomopsis* sp. (24.7%), *Neofusicoccum parvum* (23.4%) and *Xylaria venosula* (9.1%), which was attributed to their high separation rate. As did here, *Phomopsis* sp. have been reported as dominant member of the endophytic community [97]. *Phomopsis* is a dominant member of the endophytic community because it grows rapidly, thus inhibiting slow growing endophytes, which might be one of the reasons for the low number of species detected in this study [98]. Additionally, *Phomopsis* and related taxa contain important endophytic and are known to produce a series of bioactive secondary metabolites in vitro with a variety of different chemical structures [99]. However, few studies conducted on the active metabolites of *Neofusicoccum parvum*, and its antioxidant activity accounted for the highest proportion in the current study, which has never been reported in previous studies [100, 101]. Besides, *Xylaria* species are widely distributed on the temperate to the tropical zones in the terrestrial globe, and fungi of this genus have been proved to be potential sources of novel secondary metabolites, and many of them have biological activities related to drug discovery, including cytotoxic, antimalarial, and antimicrobial activities [102]. In terms of bioactivity, active extracts of DS16-1 (*Phomopsis* sp.), DR28-1 (*Phomopsis* sp.), DS35-1 (*Ceriporia lacerata*), DR41-2 (*Ceriporia lacerata*) and R46-1 (*Phomopsis* sp.) were found promising. In particular, the strain DR10-1(*Irpex lacteus*) showed wide spectrum bioactivities, suggesting that possible use of one endophyte could be a valuable candidate as new antioxidant, antimicrobial and anticancer agents.

Finally, we isolated two known compounds including indole-3-carboxylic acid and indole-3-carboxylic acid derivatives from the wide spectrum bioactive strain *I. lacteus* DR10-1. As far as we know, this was the first time that indole-3-carboxylic acid (*1*) and indole-3-carboxaldehyde (*2*) had been isolated from endophytic fungus Irpex lacteus. It was previously demonstrated that indole-3-carboxylic acid isolated from endophytic fungal strain of Epicoccum nigrum associated with Entada abyssinica had remarkable activity against Gram-negative strains (Staphylococcus aureus) with MIC values of 6.25 μg/mL [103]. This finding was consistent with literature report on the antibacterial activity of indole-3-carboxylic acid, from which a novel series of indole-3-carboxylic acid derivatives were previously reported to possess potent antibacterial activity against *Enterococcus faecalis* [104]. In addition, it has been reported that indole-3-carboxylic acid had weak cytotoxic effects on both normal and tumor cells, and its antioxidant activity is weak [103]. Recently, the indole-3-carboxylic acid (IAA) and other auxins have been shown to stimulate cell elongation, resulting in root growth initiation or an enhancement of nutritional elements absorption by the hosts [105, 106]. Besides, IAA was supposed to improve the adaptability of plant microbe interaction [107].

## Conclusions

The study provided insight into the diversity of endophytic fungal community isolated from D. chinense growing in the TGR area. This was the first report where studies on the diversity of endophytic fungus that inhabited D. chinense plant growing in the TGR area had been carried out. The data obtained showed that of the 154 endophytic fungal extracts screened for antioxidant, antimicrobial and anticancer potential. Among the 154 isolates tested, most of the endophytic fungal extracts showed abundant bioactivity. Specifically, the *I. lacteus* DR10-1 extract exhibited significant antioxidant, antimicrobial and anticancer potential. By expanding fermentation *I. lacteus* DR10-1 strain, two active secondary metabolites, indole-3-carboxylic acid (**1**) and indole-3-carboxaldehyde (**2**), were obtained, and they showed abundant biological activities. Therefore, we had for the first time reported its extract for bioactivity such as antioxidant, antimicrobial and anticancer potential. It was demonstrated that it could harbor metabolites that could serve as promising antioxidant, antimicrobial and anticancer agents.

## Supplementary information

**Additional file 1: Table S1.** Endophytic fungi from *D. chinense* and corresponding isolation frequency (IF). **Table S2.** Antioxidant activity of endophytic fungi from *D. chinense*. Three experimental replicates were taken for the assay. **Table S3.** Antimicrobial activity of endophytic fungi from *D. chinense*. The indicator organisms included gram-negative: *Escherichia coli* (EC), *Pseudomonas aeruginosa* (PA); gram-positive: *Staphylococcus aureus* (SA), *Bacillus subtilis* (BS); three pathogenic fungi *Penicillium* (P), *Aspergillus niger* (AN) and *Candida albicans* (CA). All experiments were repeated three times. **Table S4.** Anticancer activity of endophytic fungi from *D. chinense*. The indicator cells included human papillary thyroid carcinoma cell line IHH4 and human pancreatic adenocarcinoma cell line CFPAC-1. The experiments repeated for three times.

**Additional file 2: Figure S1.** Morphological characteristics and microscopic morphology of DR10–1. **Figure S2.** Neighbor-joining tree based on ITS rDNA sequence of the fungus DR10–1 and its closest ITS rDNA matches in the GenBank. **Figure S3.** ^1^H NMR spectrum of compound **1** in CD3COCD3. **Figure S4.** ^13^C and DEPT NMR spectrum of compound **1** in CD3COCD3. **Figure S5.** ^1^H NMR spectrum of compound **2** in CD3COCD3. **Figure S6.** ^13^C and DEPT NMR spectrum of compound **2** in CD3COCD3.

## Data Availability

Sequences obtained in this study were deposited in the NCBI GenBank database (For accession numbers refer Table S1 of the Additional file 1). Other datasets used and/or analyzed during the current study are available from the corresponding author upon reasonable request.

## Abbreviations

AN: *Aspergillus niger*
BS: *Bacillus subtilis*
CA: *Candida albicans*
CHCl_3_: Chloroform
*D´*: Margalef Index
DNA: DeoxyriboNucleic Acid
DPPH: 2,2′-diphenyl-b-picrylhydrazyl
*D*s: Simpson’s Diversity Index
EC: *Escherichia coli*
EtOAc: Ethyl Acetate
*H´*: Shannon-Wiener Index
HgCl: Mercuric Chloride
*IF*: Isolation Frequency
ITS: Internal Transcribed Spacer
*JC*: Jaccard Similarity Index
MeOH: Methanol
*N*: Number of Isolates
NMR: Nuclear Magnetic Resonance
P: *Penicillium*
PA: *Pseudomonas aeruginosa*
PDA: Potato Dextrose Agar
PDB: Potato Dextrose Broth
ROS: Reactive Oxygen Species
*S*: Species Richness Index
SA: *Staphylococcus aureus*
SD: Standard Deviation
TGR: Three Gorges Reservoir
Vc: Ascorbic Acid
ZI: Inhibitory Zones
