# Supplementary material for: Diversity and bioactivities of fungal endophytes from Distylium chinense, a rare waterlogging tolerant plant endemic to the Three Gorges Reservoir
Source: BMC Microbiol. 2019 Dec 10;19:278. doi: 10.1186/s12866-019-1634-0 (PMC6902458; doi:10.1186/s12866-019-1634-0)
Supplement: Supplementary file 1 — Additional file 1: Table S1. Endophytic fungi from D. chinense and corresponding isolation frequency (IF). Table S2. Antioxidant activity of endophytic fungi from D. chinense. Three experimental replicates were taken for the assay. Table S3. Antimicrobial activity of endophytic fungi from D. chinense. The indicator organisms included gram-negative: Escherichia coli (EC), Pseudomonas aeruginosa (PA); gram-positive: Staphylococcus aureus (SA), Bacillus subtilis (BS); three pathogenic fungi Penicillium (P), Aspergillus niger (AN) and Candida albicans (CA). All experiments were repeated three times. Table S4. Anticancer activity of endophytic fungi from D. chinense. The indicator cells included human papillary thyroid carcinoma cell line IHH4 and human pancreatic adenocarcinoma cell line CFPAC-1. The experiments repeated for three times. [file 12866_2019_1634_MOESM1_ESM.docx]

**Tables**

Table S1. Endophytic fungi from *D. chinense* and corresponding isolation frequency (*IF*).

Table S2. Antioxidant activity of endophytic fungi from *D. chinense*.

Table S3. Antimicrobial activity of endophytic fungi from *D. chinense*.

Table S4. Anticancer activity of endophytic fungi from *D. chinense*.

Table S1. Endophytic fungi from *D. chinense* and corresponding isolation frequency (*IF*).

| **Fungal isolates** | **Access Numbers** | **Closest relatives in NCBI** | **ITS identity (%)** | **Tissue** | **IF (%)** | **Classification** |
| --- | --- | --- | --- | --- | --- | --- |
| DR19-1 | MK172752 | *Fusarium* sp. (HM214458.1) | 99 | Root | 1.29 | *Fusarium* sp. |
| DR29-1 | MK172759 | *Fusarium equiseti* (KY963137.1) | 99 | Root | 1.29 | *Fusarium equiseti* |
| DR9-1 | MK172758 | *Penicillium ochrochloron* (KM013442.1) | 99 | Root | 1.29 | *Penicillium ochrochloron* |
| DR41-3 | MK172762 | *Diaporthales* sp. (KP006364.1) | 94 | Root | 1.29 | *Diaporthales* sp. |
| DR4-1 | MK172765 | *Phomopsis* sp. (GU066691.1) | 99 | Root | 24.67 | *Phomopsis* sp. |
| DS37 | MK172780 | *Phomopsis* sp. (KC172081.1) | 99 | Stem | 24.67 | *Phomopsis* sp. |
| DR66-1 | MK172761 | *Xylaria venosula* (KM520026.1) | 99 | Root | 9.74 | *Xylaria venosula* |
| DR48-1 | MK172763 | *Diaporthe* sp. (EF423549.2) | 99 | Root | 3.24 | *Diaporthe* sp. |
| DS15 | MK172781 | *Diaporthe* sp. (KC763081.1) | 98 | Stem | 3.24 | *Diaporthe* sp. |
| DR64-1 | MK172764 | *Lasiodiplodia theobromae* (KM508495.1) | 98 | Root | 5.19 | *Lasiodiplodia theobromae* |
| DR1-2 | MK172757 | *Neofusicoccum parvum* (KY053054.1) | 99 | Root | 23.37 | *Neofusicoccum parvum* |
| DS24-1 | MK172771 | *Neofusicoccum parvum* (KF294004.1) | 100 | Stem | 23.37 | *Neofusicoccum parvum* |
| DR32-2 | MK172753 | *Rhizoctonnia bataticola* (KT862031.1) | 99 | Root | 1.94 | *Rhizoctonnia bataticola* |
| DR58-1 | MK172760 | *Robillarda sessills* (kJ767082.1) | 94 | Root | 1.29 | *Robillarda sessills* |
| DR43-1 | MK172756 | *Coprinellus xanthothrix* (FN396102.1) | 99 | Root | 1.29 | *Coprinellus xanthothrix* |
| DR38-1 | MK172755 | *Phlebiopsis crassa* (KP135394.1) | 98 | Root | 1.29 | *Phlebiopsis crassa* |
| DR10-1 | MK172754 | *Irpex lacteus* (KT272411.1) | 99 | Root | 3.24 | *Irpex lacteus* |
| DS2-1 | MK172767 | *Fusarium oxysporum* (KF887079.1) | 99 | Stem | 2.63 | *Fusarium oxysporum* |
| DS11-1 | MK172768 | *Paraphoma* sp. (HQ914826.1) | 99 | Stem | 0.64 | *Paraphoma* sp. |
| DS9-1 | MK172770 | *Periconia* sp. (HQ130683.1) | 99 | Stem | 2.59 | *Periconia* sp. |
| DS31-2 | MK172769 | *Polyporus umbellatus* (KM185724.1) | 99 | Stem | 1.29 | *Polyporus umbellatus* |
| DSI3-1 | MK172779 | *Schizophyllum commune* (KU042974.1) | 99 | Stem | 1.94 | *Schizophyllum commune* |
| DS27 | MK172772 | *Phoma medicaginis* (FJ755260.1) | 100 | Stem | 0.66 | *Phoma medicaginis* |
| DS24 | MK172776 | *Botryosphaeria dothidea*  (KY788301.1) | 99 | Stem | 3.29 | *Botryosphaeria dothidea* |
| DSI1-2 | MK172777 | *Diaporthe longicolla*  (KR709067.1) | 99 | Stem | 1.31 | *Diaporthe longicolla* |
| DS7 | MK172773 | *Diaporthe eres*  (KY569368.1) | 99 | Stem | 0.64 | *Diaporthe eres* |
| DS29-1 | MK172775 | *Flavodon flavus*  (KT385737.1) | 99 | Stem | 1.29 | *Flavodon flavus* |
| DS3 | MK172774 | *Ceriporia lacerat*  a(JX623924.1) | 99 | Stem | 1.29 | *Ceriporia lacerata* |
| DS18-1 | MK172778 | *Irpex* sp.  (JN615247.1) | 99 | Stem | 1.29 | *Irpex* sp. |
| DS49-2 | MK172766 | *Mycorrhizal basidiomycete*  (AB176568) | 97 | Stem | 0.64 | *Mycorrhizal basidiomycete* |

Table S2. Antioxidant activity of endophytic fungi from *D. chinense*.

| Taxa | Number of activity strains (%) | Antioxidation activity (IC_50_ *μ*g/mL) | | |  |
| --- | --- | --- | --- | --- | --- |
|  |  | 10-5 | 5-3 | ≤ 3 | |
| *Botryosphaeria dothidea* | 5 (3.3) | 2 (1.3) | 3 (2.0) | 0 (0) | |
| *Ceriporia lacerata* | 2 (1.4) | 0 (0) | 1 (0.7) | 1 (0.7) | |
| *Coprinellus xanthothrix* | 2 (1.4) | 0 (0) | 2 (1.4) | 0 (0) | |
| *Diaporthe longicolla* | 2 (1.4) | 0 (0) | 2 (1.4) | 0 (0) | |
| *Diaporthales* | 1 (0.7) | 0 (0) | 1 (0.7) | 0 (0) | |
| *Diaporthe eres* | 1(0.7) | 0 (0) | 0 (0) | 1 (0.7) | |
| *Diaporthe* sp. | 4 (2.6) | 0 (0) | 4 (2.6) | 0 (0) | |
| *Fusarium equiseti* | 2 (1.4) | 1 (0.7) | 0 (0) | 1 (0.7) | |
| *Fusarium oxysporum* | 1 (0.7) | 0 (0) | 1 (0.7) | 0 (0) | |
| *Flavodon flavus* | 1 (0.7) | 0 (0) | 1 (0.7) | 0 (0) | |
| *Fusarium* sp. | 2 (1.4) | 2 (1.3) | 0 (0) | 0 (0) | |
| *Irpex lacteus* | 4 (2.6) | 1 (0.7) | 1 (0.7) | 2 (1.4) | |
| *Irpex* sp. | 2 (1.4) | 0 (0) | 2 (1.3) | 0 (0) | |
| *Lasiodiplodia theobromae* | 5 (3.2) | 2 (1.3) | 2 (1.3) | 1 (0.7) | |
| *Mycorrhizabasidiomycete* | 0 (0) | 0 (0) | 0 (0) | 0 (0) | |
| *Neofusicoccum parvum* | 21(13.6) | 5 (3.2) | 12 (7.8) | 4 (2.6) | |
| *Penicillium ochrochloron* | 2 (1.4) | 0 (0) | 1 (0.7) | 1(0.7) | |
| *Polyporus umbellata* | 2 (1.4) | 0 (0) | 1 (0.7) | 1 (0.7) | |
| *Phoma medicaginis* | 1 (0.7) | 0 (0) | 0 (0) | 1 (0.7) | |
| *Phlebiopsis crassa* | 2 (1.4) | 0 (0) | 2 (1.4) | 0 (0) | |
| *Paraphoma* sp. | 1 (0.7) | 0 (0) | 1 (0.7) | 0 (0) | |
| *Pericoma* sp. | 2 (1.4) | 0 (0) | 1 (0.7) | 1 (0.7) | |
| *Phomopsis sp* | 20(13.0) | 5 (3.2) | 13 (8.4) | 2 (1.4) | |
| *Rhizoctonia bataticolal* | 2 (1.4) | 1 (0.7) | 1 (0.7) | 0 (0) | |
| *Robillarda sessilis* | 2 (1.4) | 0 (0) | 2 (1.3) | 0 (0) | |
| *Schizphylhls commne* | 1 (0.7) | 0 (0) | 1 (0.7) | 0 (0) | |
| *Xylaria venosula* | 9 (5.8) | 2 (1.3) | 5 (3.2) | 2 (1.3) | |
| Total N (%) | 99 (64.3) | 21(13.6) | 60 (39.0) | 18 (11.7) | |

Table S3. Antimicrobial activity of endophytic fungi from *D. chinense*.

| Taxa | Number of activity strains (%) | Anti-microbial activity (%) | | | | | | |
| --- | --- | --- | --- | --- | --- | --- | --- | --- |
|  |  | Fungi pathogenic | | | Gram+ | | Gram- | |
|  |  | P | CA | AN | SA | EC | BS | PA |
| *Botryosphaeria dothidea* | 5 (3.2) | 2 (1.3) | 1 (0.6) | 1 (0.6) | 3 (2.0) | 1 (0.6) | 5 (3.2) | 5 (3.2) |
| *Ceriporia lacerata* | 2 (1.3) | 1 (0.6) | 1 (0.6) | 1 (0.6) | 2 (1.3) | 1 (0.6) | 2 (1.3) | 2 (1.3) |
| *Coprinellus xanthothrix* | 2 (1.3) | 2 (1.3) | 1 (0.6) | 1 (0.6) | 2 (1.3) | 2 (1.3) | 1 (0.6) | 1 (0.6) |
| *Diaporthe longicolla* | 1 (0.6) | 1 (0.6) | 0 (0) | 0 (0) | 2 (1.3) | 1 (0.6) | 2 (1.3) | 2 (1.3) |
| *Diaporthales* | 2 (1.3) | 0 (0) | 0 (0) | 0 (0) | 1 (0.6) | 1 (0.6) | 2 (1.3) | 1 (0.6) |
| *Diaporthe eres* | 1 (0.6) | 1 (0.6) | 1 (0.6) | 1 (0.6) | 1 (0.6) | 0 (0) | 1 (0.6) | 1 (0.6) |
| *Diaporthe* sp. | 5 (3.2) | 2 (1.3) | 1 (0.6) | 2 (1.3) | 5 (3.2) | 3 (2) | 4 (2.6) | 5 (3.2) |
| *Fusarium* sp. | 2 (1.3) | 1 (0.6) | 1 (0.6) | 1 (0.6) | 1 (0.6) | 0 (0) | 2 (1.3) | 2 (1.3) |
| *Fusarium equiseti* | 2 (1.3) | 1 (0.6) | 0 (0) | 1 (0.6) | 2 (1.3) | 1 (0.6) | 0 (0) | 2 (1.3) |
| *Fusarium oxysporum* | 4 (2.6) | 1 (0.6) | 0 (0) | 1 (0.6) | 3 (2.0) | 1 (0.6) | 1 (0.6) | 3 (2.0) |
| *Flavodon flavus* | 2 (1.3) | 0 (0) | 0 (0) | 0 (0) | 2 (1.3) | 0 (0) | 2 (1.3) | 2 (1.3) |
| *Irpex lacteus* | 6 (3.8) | 1 (0.6) | 1 (0.6) | 1 (0.6) | 4 (2.6) | 1 (0.6) | 3 (2.0) | 4 (2.6) |
| *Irpex* sp. | 2 (1.3) | 0 (0) | 1 (0.6) | 0 (0) | 2 (1.3) | 1 (0.6) | 2 (1.3) | 2 (1.3) |
| *Lasiodiplodia theobromae* | 8 (5.2) | 2 (1.3) | 0 (0) | 0 (0) | 5 (3.2) | 1 (0.6) | 7 (4.5) | 8 (5.2) |
| *Mycorrhiza basidiomycete* | 1 (0.6) | 1 (0.6) | 0 (0) | 0 (0) | 1 (0.6) | 0 (0) | 1 (0.6) | 1 (0.6) |
| *Neofusicoccum parvum* | 36 (23.4) | 12 (7.8) | 2 (1.3) | 4 (2.6) | 21 (13.6) | 8 (5.2) | 28 (18.2) | 31 (20.1) |
| *Phomopsis* sp. | 38 (24.7) | 12 (7.8) | 5 (3.2) | 11 (7.1) | 27 (17.5) | 11 (7.1) | 32 (20.8) | 37 (24.0) |
| *Penicillium ochrochloron* | 2 (1.3) | 0 (0) | 0 (0) | 0 (0) | 0 (0) | 0 (0) | 2 (1.3) | 2 (1.3) |
| *Phoma medicaginis* | 1 (0.6) | 0 (0) | 0 (0) | 0 (0) | 1 (0.6) | 1 (0.6) | 1 (0.6) | 1 (0.6) |
| *Phlebiopsis crassa* | 2 (1.3) | 0 (0) | 0 (0) | 0 (0) | 2 (1.3) | 1 (0.6) | 2 (1.3) | 2 (1.3) |
| *Paraphoma* sp. | 1 (0.6) | 0 (0) | 0 (0) | 0 (0) | 1 (0.6) | 1 (0.6) | 1 (0.6) | 1 (0.6) |
| *Pericoma* | 4 (2.6) | 2 (1.3) | 0 (0) | 1 (0.6) | 4 (2.6) | 2 (1.3) | 2 (1.3) | 4 (2.6) |
| *Polyporus umbellata* | 2 (1.3) | 1 (0.6) | 0 (0) | 2 (1.3) | 2 (1.3) | 1 (0.6) | 1 (0.6) | 2 (1.3) |
| *Rhizoctonia bataticolal* | 3 (2) | 1 (0.6) | 0 (0) | 0 (0) | 1 (0.6) | 0 (0) | 3 (2.0) | 2 (1.3) |
| *Robillarda sessilis* | 2 (1.3) | 1 (0.6) | 1 (0.6) | 0 (0) | 2 (1.3) | 1 (0.6) | 1 (0.6) | 2 (1.3) |
| *Schizphylhls commne* | 3 (2) | 0 (0) | 1 (0.6) | 0 (0) | 2 (1.3) | 1 (0.6) | 2 (1.3) | 2 (1.3) |
| Xylaria venosula | 14 (9.1) | 3 (2.0) | 1 (0.6) | 2 (1.3) | 9 (5.8) | 6 (3.9) | 10 (6.5) | 13 (8.4) |
| Total N (%) | 153 (99.4) | 48 (31.2) | 18 (11.7) | 30 (19.5) | 107 (69.5) | 46 (29.9) | 118 (76.6) | 139 (90.3) |

Table S4. Anticancer activity of endophytic fungi from *D. chinense*.

| Taxa | Number of activity strains (%) | Anticancer activity (%) | | | | | |
| --- | --- | --- | --- | --- | --- | --- | --- |
|  |  | IHH4 (IC_50_ *μ*g/mL) | | | CFPAC-1 (IC_50_ *μ*g/mL) | | |
|  |  | 50-25 | 25-10 | ≤10 | 50-25 | 25-10 | ≤10 |
| *Botryosphaeria dothidea* | 1 (0.6) | 1 (0.6) | 0 (0) | 0 (0) | 1 (0.6) | 0 (0) | 0 (0) |
| *Diaporthe longicolla* | 1 (0.6) | 0 (0) | 0 (0) | 0 (0) | 1 (0.6) | 0 (0) | 0 (0) |
| *Diaporthe eres* | 2 (1.3) | 1 (0.6) | 0 (0) | 0 (0) | 1 (0.6) | 0 (0) | 0 (0) |
| Diaporthales | 1 (0.6) | 0 (0) | 0 (0) | 0 (0) | 1 (0.6) | 0 (0) | 0 (0) |
| *Diaporthe* sp. | 1 (0.6) | 0 (0) | 1 (0.6) | 0 (0) | 0 (0) | 0 (0) | 0 (0) |
| *Fusarium oxysporum* | 2 (1.3) | 2 (1.3) | 0 (0) | 0 (0) | 1 (0.6) | 1 (0.6) | 0 (0) |
| *Fusarium equiseti* | 2 (1.3) | 0 (0) | 0 (0) | 0 (0) | 1 (0.6) | 1 (0.6) | 0 (0) |
| *Fusarium* sp. | 1 (0.6) | 1 (0.6) | 0 (0) | 0 (0) | 1 (0.6) | 0 (0) | 0 (0) |
| *Irpex lacteus* | 2 (1.3) | 1 (0.6) | 0 (0) | 1 (0.6) | 0 (0) | 1 (0.6) | 0 (0) |
| *Irpex* sp. | 2 (1.3) | 1 (0.6) | 0 (0) | 0 (0) | 1 (0.6) | 0 (0) | 0 (0) |
| *Neofusicoccum parvum* | 2 (1.3) | 1 (0.6) | 1 (0.6) | 0 (0) | 0 (0) | 1 (0.6) | 0 (0) |
| *Phoma medicaginis* | 1 (0.6) | 1 (0.6) | 0 (0) | 0 (0) | 0 (0) | 1 (0.6) | 0 (0) |
| *Penicillium ochrochloron* | 2 (1.3) | 1 (0.6) | 1 (0.6) | 0 (0) | 1 (0.6) | 0 (0) | 0 (0) |
| *Phomopsis* sp. | 4 (2.6) | 2 (1.3) | 1 (0.6) | 1 (0.6) | 2 (1.3) | 1 (0.6) | 0 (0) |
| *Xylaria venosula* | 3 (2.0) | 3 (2.0) | 0 (0) | 0 (0) | 1 (0.6) | 1 (0.6) | 0 (0) |
| Total N (%) | 27 (17.5) | 20 (13.0) | | | 19 (12.3) | | |
