# Supplementary material for: Diversity and bioactivities of fungal endophytes from Distylium chinense, a rare waterlogging tolerant plant endemic to the Three Gorges Reservoir
Source: BMC Microbiol. 2019 Dec 10;19:278. doi: 10.1186/s12866-019-1634-0 (PMC6902458; doi:10.1186/s12866-019-1634-0)
Supplement: Supplementary file 2 — Additional file 2: Figure S1. Morphological characteristics and microscopic morphology of DR10–1. Figure S2. Neighbor-joining tree based on ITS rDNA sequence of the fungus DR10–1 and its closest ITS rDNA matches in the GenBank. Figure S3. 1H NMR spectrum of compound 1 in CD3COCD3. Figure S4. 13C and DEPT NMR spectrum of compound 1 in CD3COCD3. Figure S5. 1H NMR spectrum of compound 2 in CD3COCD3. Figure S6. 13C and DEPT NMR spectrum of compound 2 in CD3COCD3. [file 12866_2019_1634_MOESM2_ESM.docx]

**Figures**

Figure S1. Morphological characteristics and microscopic morphology of DR10-1.

A. Front view of *Irpex lacteus* DR10-1 grown on PDA media 7 days;

B. Microscopic structures of *Irpex lacteus* DR10-1 grown on PDA 10 days.

Figure S2. Neighbor-joining tree based on ITS rDNA sequence of the fungus DR10-1 and its closest ITS rDNA matches in the GenBank.

Figure S3. ^1^H NMR spectrum of compound **1** in CD_3_COCD_3_.

Figure S4. ^13^C and DEPT NMR spectrum of compound **1** in CD_3_COCD_3_.

Figure S5. ^1^H NMR spectrum of compound **2** in CD_3_COCD_3_.

Figure S6. ^13^C and DEPT NMR spectrum of compound **2** in CD_3_COCD_3_.


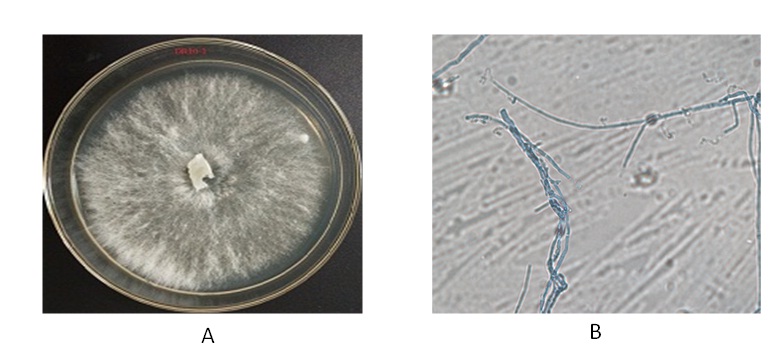


Figure S1. Morphological characteristics and microscopic morphology of DR10-1.

A. Front view of *Irpex lacteus* DR10-1 grown on PDA media 7 days;

B. Microscopic structures of *Irpex lacteus* DR10-1 grown on PDA 10 days.

**
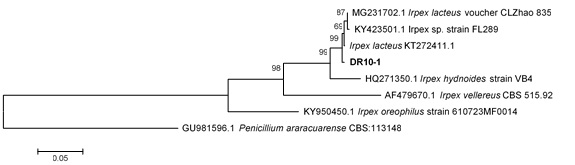
**

Figure S2. Neighbor-joining tree based on ITS rDNA sequence of the fungus DR10-1 and its closest ITS rDNA matches in the GenBank.


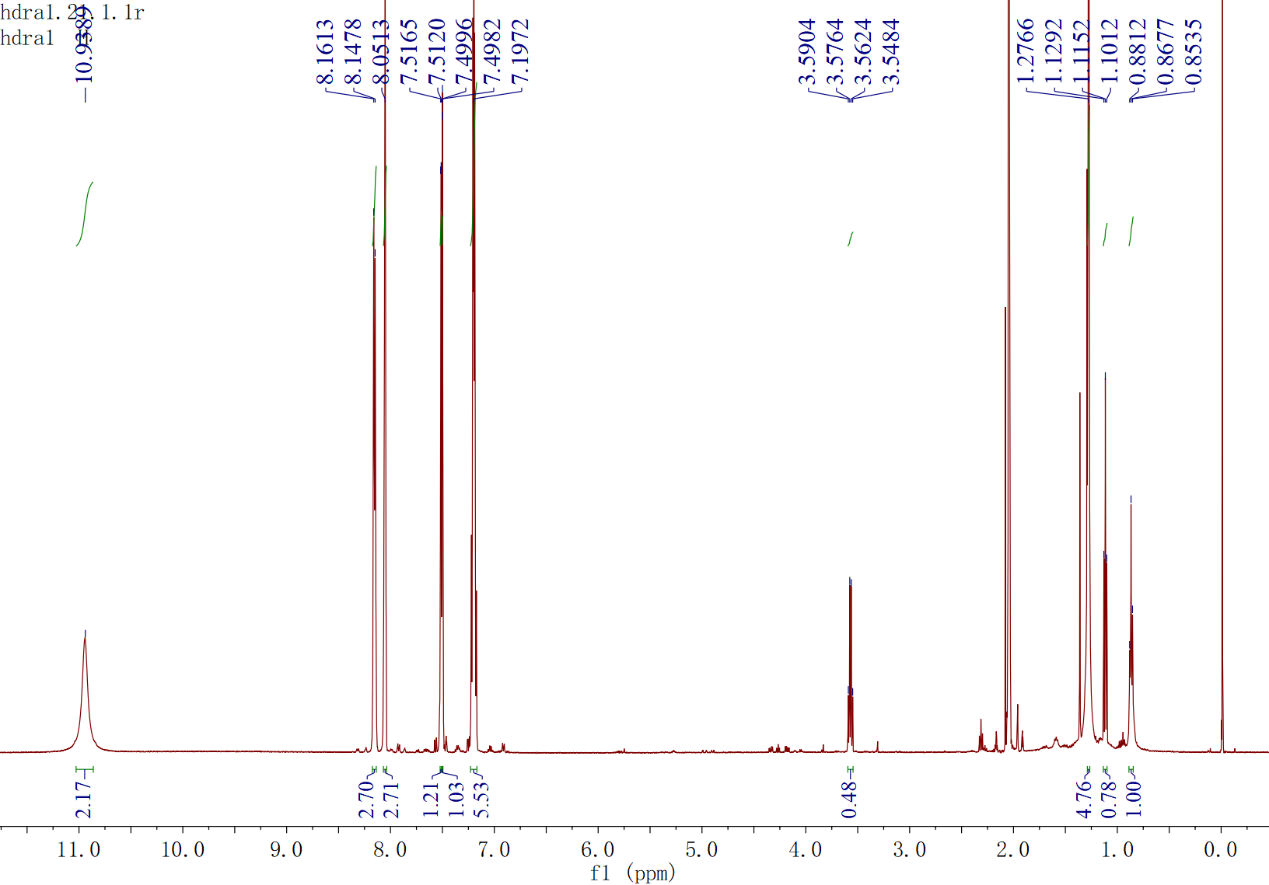


Figure S3. ^1^H NMR spectrum of compound **1** in CD_3_COCD_3_.


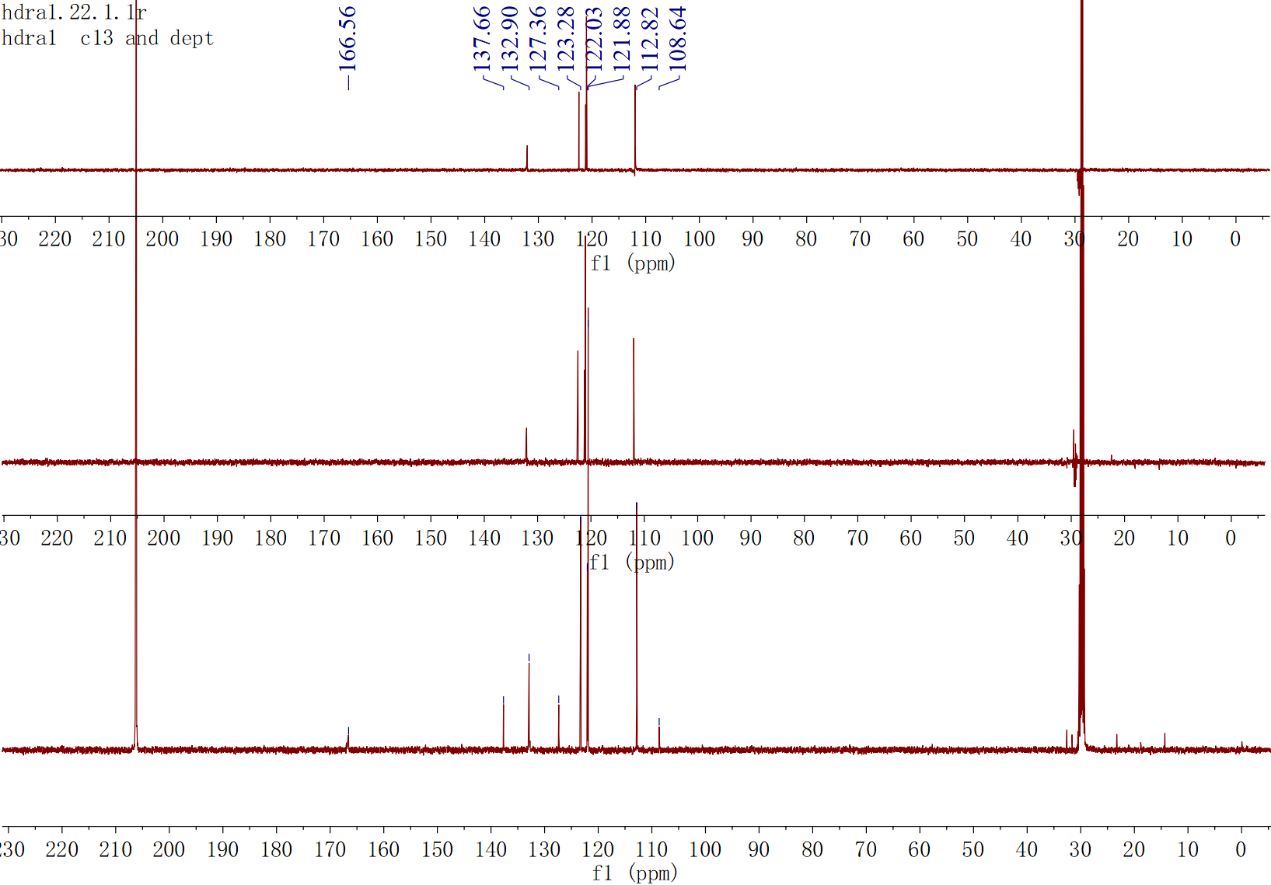


Figure S4. ^13^C and DEPT NMR spectrum of compound **1** in CD_3_COCD_3_.


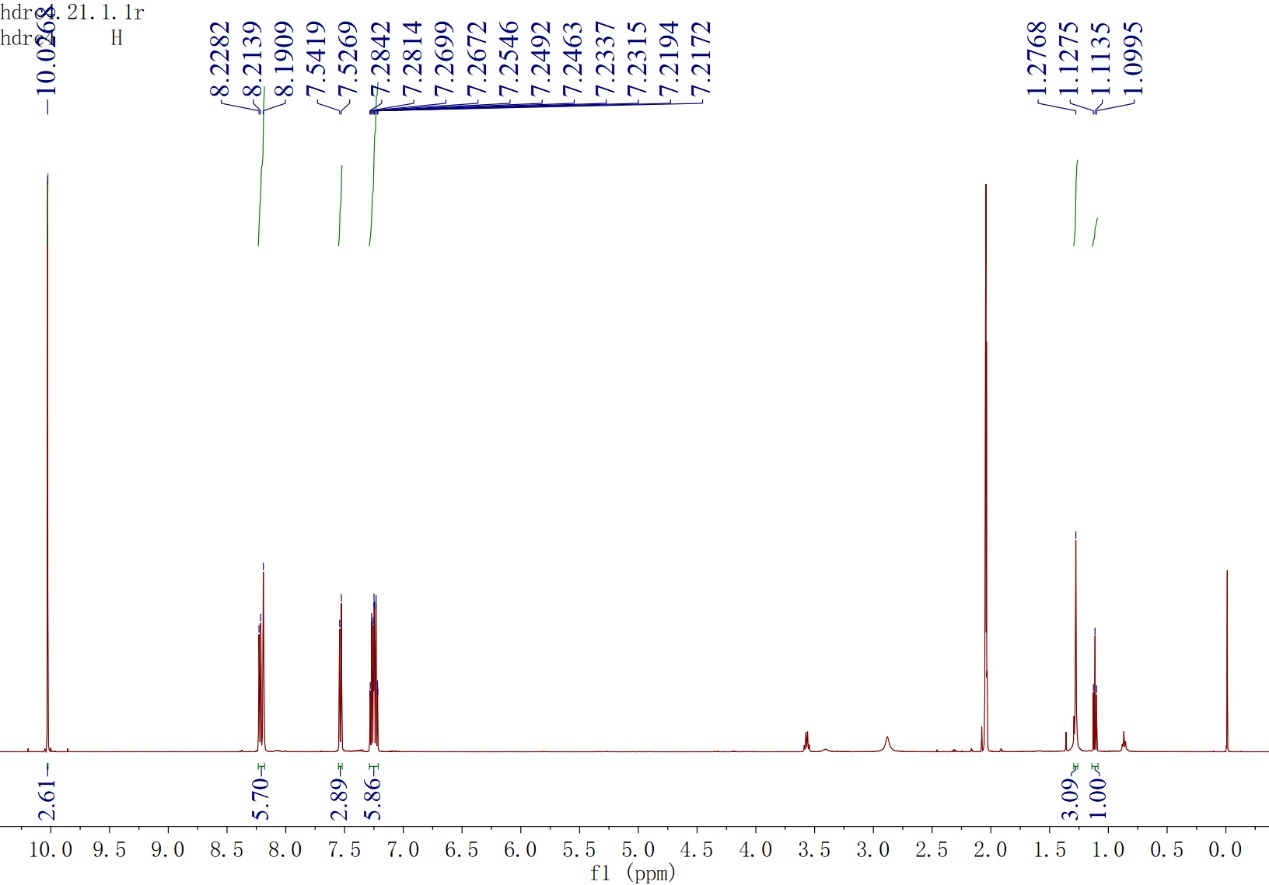


Figure S5. ^1^H NMR spectrum of compound **2** in CD_3_COCD_3_.


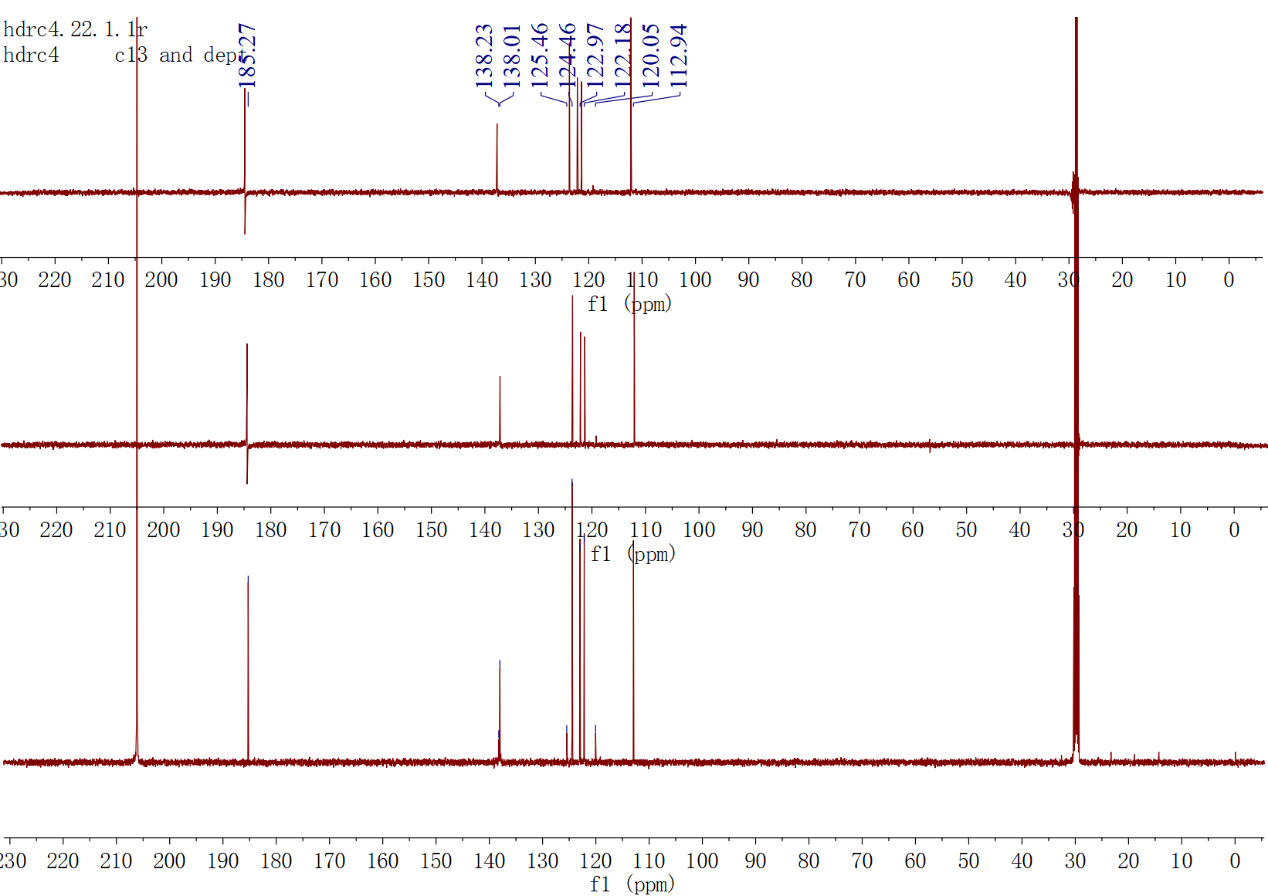


Figure S6. ^13^C and DEPT NMR spectrum of compound **2** in CD_3_COCD_3_.
